# Human Cytomegalovirus-Induces Cytokine Changes in the Placenta with Implications for Adverse Pregnancy Outcomes

**DOI:** 10.1371/journal.pone.0052899

**Published:** 2012-12-31

**Authors:** Stuart T. Hamilton, Gillian Scott, Zin Naing, Jenna Iwasenko, Beverley Hall, Nicole Graf, Susan Arbuckle, Maria E. Craig, William D. Rawlinson

**Affiliations:** 1 Virology Division, SEALS Microbiology, Prince of Wales Hospital, Sydney, Australia; 2 School of Biotechnology and Biomolecular Sciences, University of New South Wales, Sydney, Australia; 3 School of Medical Sciences, University of New South Wales, Sydney, Australia; 4 Department of Histopathology, The Children’s Hospital at Westmead, Sydney, Australia; 5 School of Women’s and Children’s Health, University of New South Wales, Sydney, Australia; 6 Institute of Endocrinology and Diabetes, The Children’s Hospital at Westmead, Sydney, Australia; The Ohio State Unversity, United States of America

## Abstract

Human cytomegalovirus (CMV) infection of the developing fetus can result in adverse pregnancy outcomes including death in utero. Fetal injury results from direct viral cytopathic damage to the CMV-infected fetus, although evidence suggests CMV placental infection may indirectly cause injury to the fetus, possibly via immune dysregulation with placental dysfunction. This study investigated the effects of CMV infection on expression of the chemokine MCP-1 (CCL2) and cytokine TNF-α in placentae from naturally infected stillborn babies, and compared these changes with those found in placental villous explant histocultures acutely infected with CMV *ex vivo*. Tissue cytokine protein levels were assessed using quantitative immunohistochemistry. CMV-infected placentae from stillborn babies had significantly elevated MCP-1 and TNF-α levels compared with uninfected placentae (*p* = 0.001 and *p* = 0.007), which was not observed in placentae infected with other microorganisms (*p* = 0.62 and *p* = 0.71) (*n* = 7 per group). Modelling acute clinical infection using *ex vivo* placental explant histocultures showed infection with CMV laboratory strain AD169 (0.2 pfu/ml) caused significantly elevated expression of MCP-1 and TNF-α compared with uninfected explants (*p* = 0.0003 and *p*<0.0001) (*n* = 25 per group). Explant infection with wild-type Merlin at a tenfold lower multiplicity of infection (0.02 pfu/ml), caused a significant positive correlation between increased explant infection and upregulation of MCP-1 and TNF-α expression (*p* = 0.0001 and *p* = 0.017). Cytokine dysregulation has been associated with adverse outcomes of pregnancy, and can negatively affect placental development and function. These novel findings demonstrate CMV infection modulates the placental immune environment *in vivo* and in a multicellular *ex vivo* model, suggesting CMV-induced cytokine modulation as a potential initiator and/or exacerbator of placental and fetal injury.

## Introduction

Human cytomegalovirus (CMV) is the leading infectious cause of congenital malformation in developed countries, with a mean global incidence of 0.64% [1]. Up to 32% of mothers with a primary CMV infection will vertically transmit virus [1]. Congenital CMV may result in the development of serious clinical sequelae [2]–[4], or at the most severe, fetal and neonatal death [5]–[7]. Reactivation of latent CMV results in at least as many adverse outcomes as primary infections [8]. Infection of the placenta, with transplacental movement of CMV across the materno-fetal interface, is a pre-requisite for infection of the fetus [9], [10]. Fetal injury results from direct viral cytopathic damage to the CMV-infected fetus [11]. However, infection can also be restricted to the placenta [6], [12], and there is increasing evidence that indirect effects of CMV through placental infection contribute to adverse pregnancy outcomes [6], [13].

Normal cytokine profiles are essential for successful establishment and maintenance of a healthy pregnancy, regulating interactions between the semi-allogeneic placenta and maternal immune system. Cytokines directly influence placental development and function including growth of anchoring and floating villi, which are essential for delivery of oxygen, nutrients and maternal IgG to the developing fetus [14]. CMV has immunomodulatory properties that alter the host immune response to infection [15] which may affect cytokine profiles within CMV-infected placentae and at the materno-fetal interface. Recent studies also indicate CMV induces a Th1 biased profile in clinically evident, primary infection of renal transplant recipients [16], and in amniotic fluid following congenital infection [17]. CMV-induced dysregulation of normal cytokine profiles during pregnancy is therefore a potential mechanism for placental dysfunction and subsequent fetal injury.

The chemokine MCP-1 (CCL2) and cytokine TNF-α have strong pro-inflammatory effects and perform vital functions in placental development, maintenance of pregnancy and protection against pathogens. MCP-1 is produced during all stages of gestation and is involved in endometrial angiogenesis, regulation of trophoblast invasion and proliferation, cellular differentiation and apoptosis [18], [19]. It is a potent chemoattractant chemokine for monocytes, macrophages and other leukocytes to sites of inflammation [20]. TNF-α is primarily expressed during early and late (rather than mid) gestation [21]. TNF-α functions during pregnancy to enhance blastocyst implantation, control trophoblast proliferation, migration and differentiation, balance trophoblast turnover and renewal, and stimulate uterine activity during parturition, as reviewed in [21]. It functions during inflammation to recruit immune effector cells, induce apoptotic cell death and has multiple functions in the septic response. While both MCP-1 and TNF-α have important roles in regulating placental development and function, aberrant expression has been associated with detrimental effects on pregnancy, which may lead to fetal injury and death [21]–[24].

The importance of cytokines in normal pregnancy, and existing evidence that CMV alters cytokine levels in various settings, led us to examine the effects of CMV infection on MCP-1 and TNF-α expression in placentae from CMV-infected stillborn babies and model this in *ex vivo* infected placental villous explant histocultures. The results show CMV infection induces changes to the immune environment within the placenta which continue after acute infection, and may be implicated in adverse pregnancy outcomes.

## Results

### MCP-1 and TNF-α Expression is Elevated in CMV-infected Placentae from Stillborn Babies

**Figure 1:**
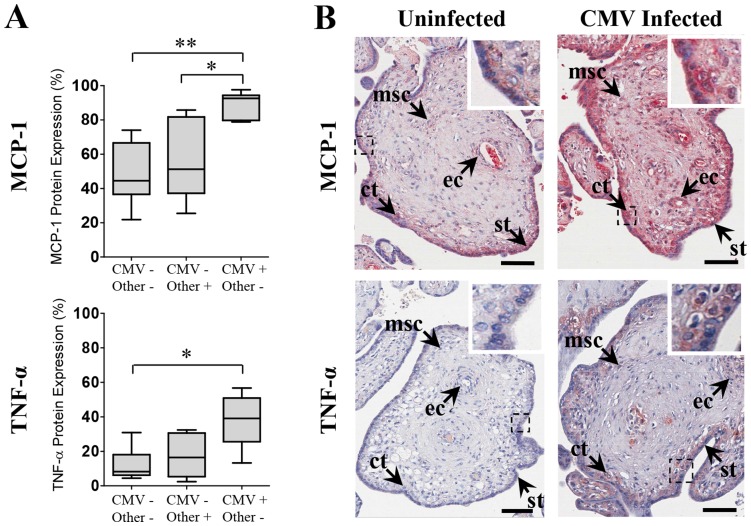
MCP-1 and TNF-α expression is elevated in CMV-infected placentae from stillborn babies. (A) MCP-1 and TNF-α expression in uninfected (CMV−/Other−), other microorganism-infected (CMV−/Other+) and CMV-infected (CMV+/Other−) placentae from stillborn babies. Data presented as box plots with median value, Q1, Q3 and range. Significant differences between groups were determined with the Mann-Whitney U test and threshold for significance adjusted to account for multiple comparisons using Bonferroni’s correction; *p<0.0167, **p<0.003. (B) Representative images of immunohistochemical localisation of MCP-1 and TNF-α protein (red staining) in uninfected and CMV-infected placental tissue from stillborn babies. In chorionic villi, cytokine localised in syncytiotrophoblast (st) and cytotrophoblast (ct) cells, mesenchymal stromal cells (msc) and endothelial cells of fetal capillaries (ec). Scale bars represent 70 µm.

MCP-1 and TNF-α were elevated in CMV-infected placentae delivered from infected women, compared with placentae infected with other microorganisms and uninfected placentae (Figure 1). In the naturally infected placentae, MCP-1 and TNF-α expression were significantly elevated in CMV-infected compared with uninfected placentae (*p* = 0.001 and *p* = 0.007 respectively) (Figure 1A). MCP-1 levels were also significantly elevated in CMV-infected placentae compared with placentae infected with other microorganisms (*p* = 0.011), while TNF-α levels were not significantly different (*p* = 0.038) using the highly conservative Bonferroni-corrected threshold for significance (*p*<0.0167). No significant difference was found in MCP-1 or TNF-α levels for placentae infected with other microorganisms compared with uninfected placentae (*p* = 0.62 and *p* = 0.71 respectively), suggesting this was a CMV-specific effect.

MCP-1 and TNF-α protein localised in syncytiotrophoblast, cytotrophoblast, mesenchymal and endothelial cells of chorionic villi in CMV-infected placentae, placentae infected with other microorganisms and uninfected placentae from stillborn babies (Figure 1B). Staining was not observed in any control sections incubated with IgG isotype negative control primary antibody in parallel with experimental sections (data not shown).

### Modelling *in vivo* CMV Infection of the Placenta Using *ex vivo* Placental Explant Histocultures

Term placental villous explants remain viable for up to 21 days in *ex vivo* culture and are more readily infected indirectly via an underlying feeder cell monolayer as opposed to direct virus inoculation [25]. In this study, the majority of chorionic villi remained morphologically normal, and expression of placental hormones placental lactogen and chorionic gonadotropin continued to be detected by immunofluorescence at day 12 of explant culture, consistent with continuing explant viability. However, isolated syncytiotrophoblast detachment was observed in some peripheral villi of CMV- and mock-infected explants, presumably as a consequence of placental dissection, as has been previously observed [26]. Cytomegalic cells were also readily observed in chorionic villi of CMV-infected explants which were not observed in uninfected control explants (data not shown).

**Figure 2:**
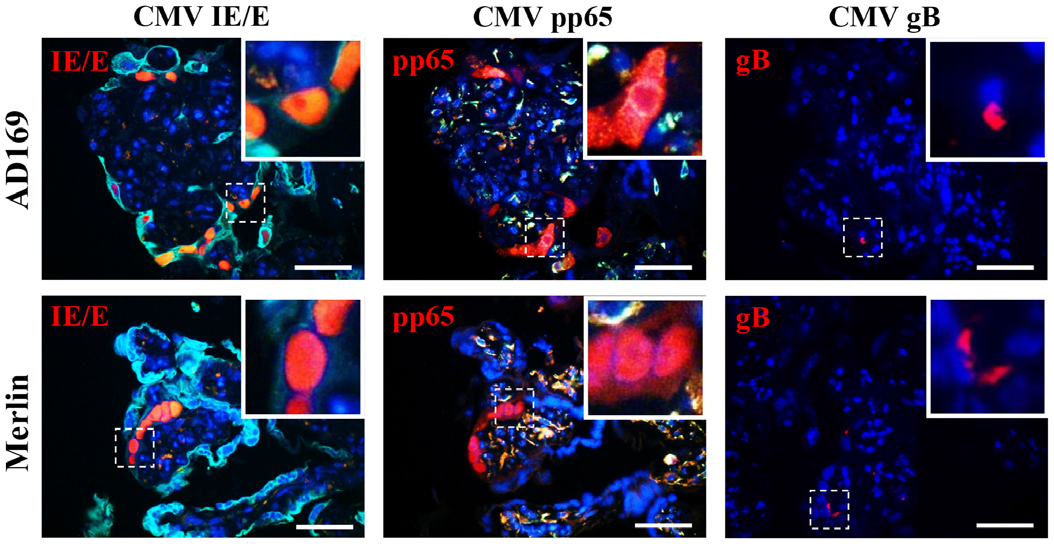
CMV productively infects placental villous explants. Productive AD169 and Merlin infection of villous explants was determined by staining for CMV immediate early/early (IE/E), early/late (pp65) and late (gB) protein. Representative images are of AD169 and Merlin infected villous explants 12 days post inoculation. Scale bars represent 50 µm.

Productive CMV infection of villous explants was demonstrated using immunofluorescence detection of CMV immediate early/early (IE/E), pp65 early/late and gB late protein in consecutive histological sections (Figure 2). Staining for CMV antigens was negative in mock-infected explants, and no staining was observed in sections incubated with IgG isotype negative control primary antibody (data not shown).

**Figure 3:**
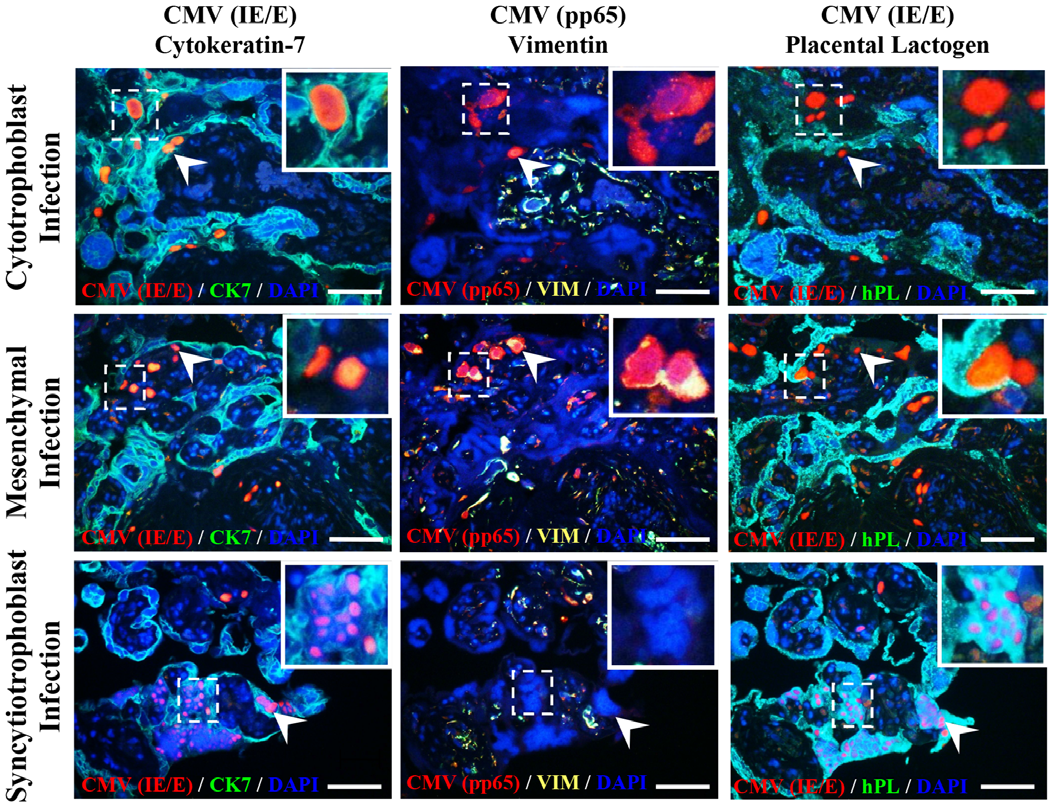
CMV actively replicates in cytotrophoblast and mesenchymal cells, but not syncytiotrophoblasts, of placental villous explants. CMV laboratory strain AD169 and wild-type Merlin infected syncytiotrophoblasts (CK7+/VIM−/hPL+), cytotrophoblasts (CK7+/VIM−/hPL−) and mesenchymal cells of the villous stroma (CK7−/VIM+/hPL−) as determined by staining for CMV immediate early/early protein. Active replication (CMV IE/E+, pp65+) was observed in both cytotrophoblasts and mesenchymal cells but not syncytiotrophoblasts (CMV IE/E+, pp65−) (inserts and arrow heads). Representative images are of 4 µm consecutive histological sections of AD169 infected villous explants 12 days post inoculation. No difference in cellular tropism was observed between AD169 and Merlin strains. Scale bars represent 100 µm.

Villous explants infected with AD169 or Merlin showed virion proteins in syncytiotrophoblasts, cytotrophoblasts and mesenchymal cells of chorionic villi. Cytotrophoblast and mesenchymal cells were permissive for productive viral infection as determined by immunofluorescence detection of CMV IE/E and pp65 antigens in adjacent nuclei of consecutive histological sections. However, CMV IE/E protein was rarely detected, and pp65 never detected, in syncytiotrophoblast nuclei of CMV-infected explants (*n* = 50), suggesting abortive infection or (less likely) significantly delayed growth kinetics within these cells (Figure 3). Furthermore, there were two distinct staining patterns observed for CMV gB protein in CMV-infected explants - limited punctate staining, or diffuse gB accumulation within cell cytoplasm, representing non-replicating viral particles and late productive infection respectively [10]. Punctate staining was observed in syncytiotrophoblast, cytotrophoblast and mesenchymal cell cytoplasm, however, the diffuse accumulation of gB protein was only detected in cytotrophoblast and mesenchymal cells and not in syncytiotrophoblasts (data not shown).

**Figure 4:**
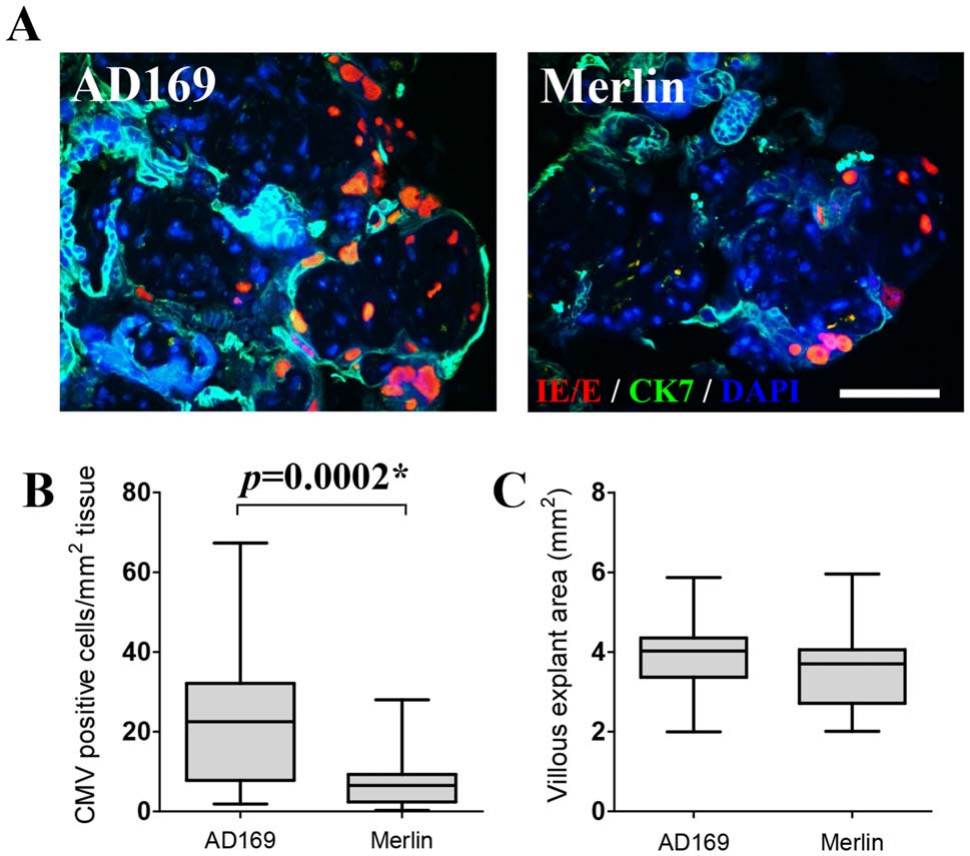
CMV infection is significantly greater in AD169- compared with Merlin-infected placental villous explants. (A) Representative images of CMV immediate early/early (IE/E) antigen detection in AD169- (left) and Merlin-infected (right) placental villous explants 12 days post inoculation (CK7; Cytokeratin-7). Scale bar represents 70 µm. (B) Number of cells per mm^2^ of villous explant tissue expressing CMV IE/E protein in AD169- compared with Merlin-infected explants. (C) No significant differences in villous explant area (mm^2^) were observed between the CMV-infected explant groups (*p* = 0.1). Data presented as box plots with median value, Q1, Q3 and range. Significant differences between groups (denoted as *) were determined using a one-tailed Spearman’s correlation.

While both AD169- and Merlin-infected explants showed productive infection within the explant tissue, significantly lower levels of cell associated viral protein was observed in Merlin-infected compared with AD169-infected explants (*p = *0.0002) (Figure 4A and 4B). This is consistent with the different multiplicities of infection (MOI) used for the CMV AD169 and Merlin strains (0.2 pfu/ml and 0.02 pfu/ml respectively). No significant difference in villous explant area (mm^2^) was observed between the AD169- and Merlin-infected explants (*p* = 0.1) (Figure 4C).

### CMV Infection of *ex vivo* Placental Explants Induced Upregulation of MCP-1 and TNF-α Expression in Response to Infection

**Figure 5:**
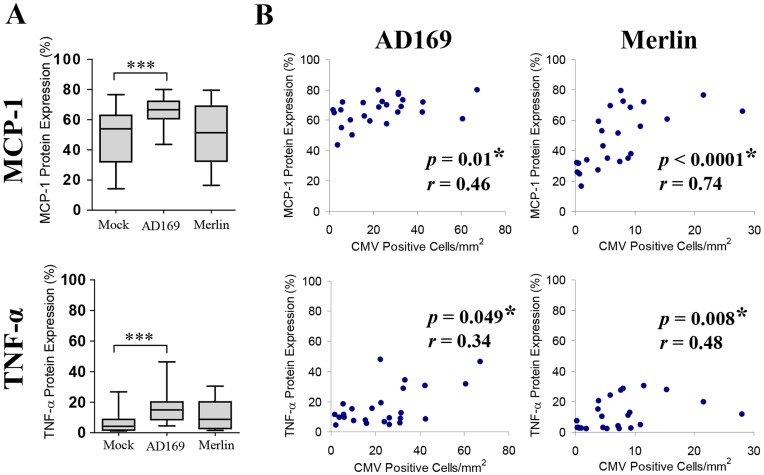
CMV infection of placental villous explants results in upregulation of MCP-1 and TNF-α expression. (A) MCP-1 and TNF-α expression in mock-infected, AD169-infected (0.2 pfu/ml) and Merlin-infected (0.02 pfu/ml) villous explants. Data presented as box plots with median value, Q1, Q3 and range. Significant differences between groups were determined with the Mann-Whitney U test and threshold for significance adjusted to account for multiple comparisons using Bonferroni’s correction; *p<0.025, **p<0.005 and ***p<0.0005. (B) Correlation between degree of CMV placental explant infection with corresponding MCP-1 and TNF-α expression. MCP-1 and TNF-α protein expression was plotted against the number of cells expressing CMV immediate early/early (IE/E) protein per mm^2^ of AD169 and Merlin infected villous explant tissue. Significant correlations (denoted by *) were determined by two-tailed nonparametric spearmans correlation with trend plotted as straight lines.

Compared with uninfected placental villous explants, AD169 infection of explants at an MOI of 0.2 pfu/ml resulted in significantly elevated expression of MCP-1 and TNF-α (*p* = 0.0003 and *p*<0.0001 respectively) (Figure 5A). Merlin infection at a tenfold lower MOI of 0.02 pfu/ml did not show a significant difference in MCP-1 or TNF-α expression compared with mock-infected placental explants (*p* = 0.82 and *p* = 0.054 respectively). Given the lack of significance for CMV Merlin strain compared with mock-infected explants and limited amount of infection observed, we further explored the relationship between CMV infection and upregulation of MCP-1 and TNF-α expression by plotting the number of CMV positive cells/mm^2^ of explant tissue against the corresponding cytokine response (Figure 5B). Merlin-infected explants showed a significant positive correlation between degree of explant infection and MCP-1 and TNF-α expression (*p*<0.0001 and *p* = 0.008 respectively) demonstrating Merlin infection of placental explants was associated with upregulation of MCP-1 and TNF-α in response to clinical infection. AD169 showed a similar significant positive correlation for degree of explant infection and MCP-1 and TNF-α expression (*p* = 0.01 and *p* = 0.049 respectively).

Cytokine protein localised in chorionic villi of the placental explants in a manner analogous to localisation seen in clinically infected and uninfected placental tissue from stillborn babies (Figure 1B).

## Discussion

Expression of the pro-inflammatory chemokine MCP-1 and cytokine TNF-α was elevated in CMV-infected placentae from stillborn babies, and this was reflected in results from acute infection of a primary multicellular placental model. CMV-induced upregulation of MCP-1 and TNF-α expression is consistent with previous studies investigating CMV-induced immune modulation in other settings including renal transplants [16], cell culture studies [27], [28] (including our own unpublished findings), and our recent observations in amniotic fluid of CMV-infected pregnant women [17]. This may be a more general feature of CMV infection *in vivo* or may be a feature of immune compromised populations.

The congruence between elevated MCP-1 and TNF-α in amniotic fluid of CMV-infected pregnant women and tissue fixed cytokine in both CMV-infected stillbirth placentae and *ex vivo* placental explant histocultures suggests the amniotic fluid cytokine changes are related to placental changes. The placental source of these cytokines with pro-inflammatory effects are likely to be placental trophoblasts, fetal macrophages (Hofbauer cells) and stromal cells [29], [30] consistent with our immunohistochemical findings (Figure 1B).

While AD169 infection of placental explants produced a significant MCP-1 and TNF-α response compared with mock-infected explants, these results were not reflected with the wild-type Merlin strain. This is primarily attributed to the differences in multiplicity of infection (MOI) used between the AD169 and Merlin strains (0.2 and 0.02 pfu/ml respectively), due to the relationship between MOI used to initially infect the underlying cell monolayer and subsequent number of cells expressing CMV antigen in the explant tissue (Figure 4). In support of this, there was a consistent, significant positive correlation between Merlin infection and cytokine upregulation (Figure 5B). The slower growth kinetics of Merlin compared with AD169 is responsible for the limitation in achieving a high enough inoculation dose for Merlin [31], and may also be a contributing factor in the cytokine expression differences observed for the two viruses.

Elevated MCP-1 expression has been associated with adverse pregnancy outcomes including preeclampsia [32], intrauterine growth restriction, spontaneous abortion, pregnancy loss [22] and preterm delivery [33]. All of these outcomes (excepting preeclampsia) have also been shown to result from congenital CMV infection in previous clinical studies. Increases in macrophage populations aberrantly activated by human lipopolysaccharide are associated with decreased cytotrophoblast invasion of uterine decidua [23] and apoptosis of the syncytiotrophoblast layer [34] *in vitro*. Given the potent effects MCP-1 has on macrophage activation and chemotaxis, CMV-induced MCP-1 increases may similarly affect cytotrophoblast invasiveness of the uterine decidua during placental development and syncytiotrophoblast function during gestation. Furthermore, elevated MCP-1 levels could assist virus dissemination through recruitment of monocytes and macrophages to sites of infection.

Elevated TNF-α has been linked to adverse effects on pregnancy and placental function including embryonic death, spontaneous abortion and preterm labor in amniotic fluid of animal (mouse) models [24], [35], [36], reduced migration and invasion capabilities of cytotrophoblast cells *in vitro* in placental explant and trophoblast cell cultures [37], [38], apoptosis of syncytiotrophoblasts [34], [39], diminished trophoblast syncytialisation [21], degradation of decidual extracellular matrix [37], [40], and diminished expression of hormones such as hCG in cell culture models [41]. Normal cellular processes for these functions are critical for successful establishment and maintenance of pregnancy, and the reductionist and animal models described show TNF-α has a significant effect on most of them in these models.

The non-permissive nature of syncytiotrophoblasts to CMV infection found here in acute infection of term placental explants *ex vivo* is consistent with previous findings in both clinical and *ex vivo* placental tissue [10], [42]. Similar results have been reported by other groups for placental infection by herpes simplex virus [43], adenovirus [44] *Listeria monocytogenes* and *Toxoplasma gondii* [26]. Our results further support the hypothesis of Robbins *et al.* [26] and others [42], [43] that the syncytiotrophoblast layer acts as a general barrier to placental infections, thereby requiring infectious agents to employ specific adaptive strategies to cross the materno-fetal interface and disseminate through the fetal tissues.

These data suggest CMV-induced placental upregulation of cytokines with pro-inflammatory effects such as MCP-1 and TNF-α could lead to fetal injury, and in some cases fetal demise, via several different mechanisms including: i) placental insufficiency resulting from shallow placentation [23], [37], [38] and/or diminished function of the syncytiotrophoblasts in nutrient and gas exchange across the materno-fetal interface [21], [39]; ii) increased materno-fetal virus transmission and dissemination due to compromised integrity of the syncytiotrophoblast barrier to placental infection [26], [34], [39], [43]; iii) adverse downstream effects on other cellular proteins critical for successful pregnancy such as placental hormones [41], matrix metalloproteinases [45] and adhesion molecules [39]; and/or iv) loss of immune tolerance to the semi-allogeneic placenta resulting from a CMV-induced Th1 shift in placental cytokine profiles during gestation [17]. While our data do not specifically address these mechanistic questions, they do provide novel insights into CMV-induced immune modulation within both naturally infected placentae and an *ex vivo* placental explant model.

The placental explant histocultures provide a primary multicellular model of the human placenta that more accurately reflects the *in vivo* placental environment than other model systems, such as cell culture monolayers and animal models. Continuous placental cell lines lack gene expression profiles of their primary cell counterparts due to cellular transformation [46], while primary placental cell monocultures lack the protein network interactions between different cell types found in a multicellular system. Animal models provide useful whole-animal models, with fetal outcome data available, but do have the limitations of significant differences in placental development, morphology and function between species [47], and the necessity of using species-specific strains of CMV in these models [48], overcome to some extent by the use of humanised animals [49]. Here we demonstrate that placental explant histocultures provide a unique and robust research tool for future investigations into the placental immune response to infection and subsequent effects on placental function.

This novel study is the first to specifically investigate CMV-induced cytokine changes in placentae from stillborn babies and utilise placental explant histocultures to model *in vivo* immunological changes in response to *in vitro* microbiological infection. These data suggest a possible indirect mechanism for CMV-induced fetal injury via placental upregulation of cytokines and chemokines with pro-inflammatory effects. Clearly the placenta responds to CMV infection acutely *in vitro*, and chronically *in vivo* with a Th1 biased response, on the basis of our observations here in the placenta, and elsewhere in the amniotic fluid [17]. This suggests these cytokine changes may be used as markers of adverse pregnancy outcomes from CMV infection, and potentially as therapeutic targets.

## Methods

### Ethical Statement

This study was conducted according to the principles expressed in the Declaration of Helsinki. Approval for this study was obtained from South Eastern Sydney and Illawarra Area Health Service Human Research Ethics Committee (04-210 [11 August 2005] and 09-012 [19 June 2009]), and site-specific approval was provided by the Children’s Hospital at Westmead (MR 2009-04-03 [27 February 2009]) and the Prince of Wales Hospital (09-G-019 [21 July 2009]) in Sydney, Australia. All patients provided written informed consent for the collection of samples and subsequent analysis.

### Stillbirth Case Series and Specimen Collection

Specimens were selected from a retrospective case series of formalin fixed, paraffin-embedded placentae collected from stillborn infants at post-mortem examination during the period January 2005 through December 2006. Bacterial cultures and multiplex PCR were performed for all specimens as previously described [6]. Placental specimens were classified as: i) CMV-infected placentae, ii) placentae infected with other microorganisms (including enterovirus, herpes simplex virus, human herpes virus 8, human herpes virus 7, *Mycoplasma hominis*, *Mycoplasma genitalium*, or Group B *Streptococcus*), and iii) uninfected placentae (n = 7 per group). Specimens were matched for gestational age between groups with a median age within each group of 35 weeks (range: 21–42 weeks).

### Viruses

Wild-type CMV strain Merlin (UL128+, RL13−) was derived from a Merlin-BAC recombinant, pAL1120, kindly provided by Richard Stanton (University of Cardiff). DNA was transfected and propagated in ARPE-19 cells (ECACC) in DMEM/F12+GlutaMAX (Invitrogen) +5% fetal bovine serum (FBS; Bovagen) and 100 U/ml penicillin G, 100 ug/ml streptomycin and 29.2 ug/ml L-glutamine (1×PSG, Invitrogen), with one passage in MRC-5 cells to increase viral titre, as previously described [50]. Merlin was propagated in ARPE-19 cells following single passage in MRC-5 fibroblasts demonstrating supernatant virus from Merlin-infected MRC-5 cells retained epithelial cell tropism. CMV laboratory strain AD169 (ATCC) was cultured in MRC-5 fibroblasts (ECACC) with MEM (Invitrogen) supplemented with 2% FBS and PSG. The titre of virus stocks was quantified using standard plaque assay.

### Placental Villous Explant Histocultures

Placental tissue was cultured *ex vivo*, and infected with CMV, using methods similar to those previously described to examine placental cytokines [51] and CMV infection of placentae *ex vivo* [25] with some minor modifications. Four term placentae were collected with consent from women undergoing elective Caesarean section delivery who had a healthy pregnancy and were not in labor. The uninfected status of each placenta used in experiments was verified by multiplex PCR [6].

Five days prior to collection of placentae, 6-well culture plates with 90% confluent monolayers of MRC-5 fibroblasts were inoculated with CMV wild-type strain Merlin or laboratory adapted strain AD169 (n = 6) at a multiplicity of infection (MOI) of 0.02 or 0.2 pfu/ml, respectively, in MEM supplemented with 10% FBS and 1×PSG. Mock infected (media only) cultures were established concurrently (n = 6). Following centrifugation of plates at 770×g for 30 min and one hour incubation at 37°C with 5% CO2, supernatant was removed and replaced with 2 ml RPMI-1640 (Invitrogen, USA) supplemented with 15% FBS and 1×PSG. Virus- and mock-infected MRC-5 cultures were re-incubated at 37°C with 5% CO2 for four days. An additional 3 ml of RPMI-1640 media supplemented with 15% FBS and 1% PSG was added to the CMV- and mock-infected MRC-5 cultures one hour prior to collection of placentae.

Placentae were collected and dissected within three hours of delivery to obtain placental cotyledons. The cotyledons were washed in three changes of 0.9% sodium chloride solution supplemented with 1×PSG and cut to obtain 3–5 mm^2^ chorionic villi explants. Explants were placed on sterile collagen sponge gels (Pharmacia and Upjohn, USA) with four-five villous explants per sponge. Sponge gels with villous explants were added to the CMV- and mock-infected MRC-5 culture wells (one sponge per well) and explants cultured at the liquid-air interface bathed in culture media. There was no direct contact between the sponge gels and underlying MRC-5 monolayer. The placental explant histocultures were incubated for four days at 37°C with 5% CO2 with explants basted in 1 ml volumes of culture media several times each day. At day five, the explants and collagen sponge gels were transferred to fresh 6-well plates containing 5 ml fresh culture media, but with no underlying cell monolayer, and incubated for a further seven days. At day nine of explant culture, 3 ml of fresh culture media was again added to the explant histocultures. Histocultures were gently agitated each day to assist nutrient and virus dissemination through the explant tissue. The mock-, AD169- and Merlin-infected villous explants (n = 25 per group) were harvested at day 12 of explant culture, fixed in 10% buffered formalin and paraffin embedded.

### Cytokine Protein Detection by Immunohistochemistry

De-paraffinised and rehydrated 4 µm placental sections were incubated with 0.5% Tween20 in PBS for 10 min, peroxidase blocking reagent (Dako) for 15 min, then 2% bovine serum albumin (BSA) in PBS for 30 min. Cytokine protein was detected by incubating sections with mouse monoclonal anti-human MCP-1 (R&D Systems; 1∶30 dilution) or mouse monoclonal anti-human TNF-α (R&D Systems; 1∶20 dilution) primary antibody for 90 min, HRP-labelled polymer anti-mouse secondary antibody (Dako) for 40 min followed by 3-amino-9-ethylcarbazole (Dako) for 15 min. Sections were counterstained with Mayer’s haematoxylin (Dako) and sealed with CC/Mount (Sigma). Infected and uninfected sections incubated with IgG isotype primary antibody (Dako) served as a negative control and were included with each experiment in parallel. Stained sections were digitally scanned using the ScanScope XT Slide Scanner (Aperio) and analysed using ImageScope area quantitation software (Positive pixel count v9; Aperio). Percentage of cytokine protein expression was the number of positive pixels detected divided by total number of pixels present in 20 fields of view for each stillbirth placenta and one entire section for each villous explant.

### Co-localisation of Viral and Cellular Proteins by Dual Immunofluorescence

De-paraffinised and rehydrated 4 µm placental sections were subjected to antigen retrieval using Tris-EDTA buffer pH 9.0 for 20 min at 95°C followed by 30 min incubation at room temperature. Non-specific staining was blocked with 2% BSA for 30 min. CMV was detected by 60 min incubation with mouse monoclonal anti-CMV Immediate Early and Early (IE/E) antibody (Clones DDG9 and CCH2; Dako; 1∶100 dilution), mouse monoclonal anti-CMV pp65 antibody (Abcam; 1∶200 dilution), or mouse monoclonal anti-CMV gB antibody (Abcam; 1∶200 dilution) followed by 40 min incubation with Alexa Fluor 594 IgG goat anti-mouse secondary antibody (Invitrogen; 1∶1000 dilution). Sections were then incubated with primary antibodies for mouse monoclonal anti-human cytokeratin-7 (Abcam; 1∶100 dilution), mouse monoclonal anti-human vimentin (ICN; 1∶20 dilution) or rabbit polyclonal anti-human placental lactogen (Abcam; 1∶200 dilution) for 60 min, followed by 40 min incubation with Alexa Fluor 488 IgG_1_ goat anti-mouse or Alexa Fluor 488 IgG donkey anti-rabbit secondary antibodies (Invitrogen; 1∶1000 dilution). ProLong Gold Antifade Reagent containing DAPI (Invitrogen) was added to each slide and mounted with a coverslip. Infected and uninfected sections incubated with IgG isotype primary antibody served as a negative control. Imaging of stained tissue sections were carried out using a Nikon Eclipse E400 light microscope with a Y-FL Epi Fluorescence attachment and a DS camera control unit DS-L2, DS camera head DS-Fi1 (Nikon).

### Correlation between Viral Infection and Cytokine Expression

A representative section from each AD169-infected and Merlin-infected explant was stained for CMV IE/E protein as described. The total number of CMV IE/E positive cells for each explant was calculated and divided by total area of the section (mm^2^) to give a normalised value of CMV positive cells per mm^2^ tissue. Explant section area was determined by the Aperio ImageScope area quantitation software. The number of CMV positive cells/mm^2^ tissue was then plotted against the corresponding MCP-1 or TNF-α protein expression results for each infected explant.

### Statistical Analysis

Data were not normally distributed and so non-parametric tests were used for analyses. Differences between groups were assessed using the Mann-Whitney U test. Bonferroni’s correction was applied to adjust for multiple comparisons where applicable. The Bonferroni-corrected threshold for significance was calculated by dividing the conventionally set level of significance (*p<*0.05) by the number of comparisons. The association between two continuous variables was assessed using a one-tailed Spearman’s correlation coefficient test. Data were analysed using SPSS Statistics (Version 20, IBM Corporation).

## References

[1] Kenneson A, Cannon MJ (2007) Review and meta-analysis of the epidemiology of congenital cytomegalovirus (CMV) infection. Rev Med Virol 17: 253–276. 17579921 10.1002/rmv.535

[2] Nigro G, Adler SP (2011) Cytomegalovirus infections during pregnancy. Curr Opin Obstet Gynecol 23: 123–128. 21157339 10.1097/GCO.0b013e328342f1f6

[3] Boppana SB, Pass RF, Britt WJ, Stagno S, Alford CA (1992) Symptomatic congenital cytomegalovirus infection: neonatal morbidity and mortality. Pediatr Infect Dis J 11: 93–99. 1311066 10.1097/00006454-199202000-00007

[4] Dollard SC, Grosse SD, Ross DS (2007) New estimates of the prevalence of neurological and sensory sequelae and mortality associated with congenital cytomegalovirus infection. Rev Med Virol 17: 355–363. 17542052 10.1002/rmv.544

[5] Gaytant MA, Steegers EA, Semmekrot BA, Merkus HM, Galama JM (2002) Congenital cytomegalovirus infection: review of the epidemiology and outcome. Obstet Gynecol Surv 57: 245–256. 11961482 10.1097/00006254-200204000-00024

[6] Iwasenko JM, Howard J, Arbuckle S, Graf N, Hall B, et al (2011) Human cytomegalovirus infection is detected frequently in stillbirths and is associated with fetal thrombotic vasculopathy. J Infect Dis 203: 1526–1533. 21592980 10.1093/infdis/jir121

[7] Stagno S, Pass RF, Cloud G, Britt WJ, Henderson RE, et al (1986) Primary cytomegalovirus infection in pregnancy. Incidence, transmission to fetus, and clinical outcome. JAMA 256: 1904–1908. 3020264

[8] Wang C, Zhang X, Bialek S, Cannon MJ (2011) Attribution of congenital cytomegalovirus infection to primary versus non-primary maternal infection. Clin Infect Dis 52: e11–13. 21288834 10.1093/cid/ciq085

[9] Fisher S, Genbacev O, Maidji E, Pereira L (2000) Human cytomegalovirus infection of placental cytotrophoblasts in vitro and in utero: implications for transmission and pathogenesis. J Virol 74: 6808–6820. 10888620 10.1128/jvi.74.15.6808-6820.2000 PMC112198

[10] Maidji E, Genbacev O, Chang HT, Pereira L (2007) Developmental regulation of human cytomegalovirus receptors in cytotrophoblasts correlates with distinct replication sites in the placenta. J Virol 81: 4701–4712. 17314173 10.1128/JVI.02748-06 PMC1900158

[11] Gabrielli L, Bonasoni MP, Lazzarotto T, Lega S, Santini D, et al (2009) Histological findings in foetuses congenitally infected by cytomegalovirus. J Clin Virol 46 Suppl 4S16–21. 10.1016/j.jcv.2009.09.026 19879801

[12] Trincado DE, Munro SC, Camaris C, Rawlinson WD (2005) Highly sensitive detection and localization of maternally acquired human cytomegalovirus in placental tissue by in situ polymerase chain reaction. J Infect Dis 192: 650–657. 16028134 10.1086/431999

[13] Maidji E, Nigro G, Tabata T, McDonagh S, Nozawa N, et al (2010) Antibody treatment promotes compensation for human cytomegalovirus-induced pathogenesis and a hypoxia-like condition in placentas with congenital infection. Am J Pathol 177: 1298–1310. 20651234 10.2353/ajpath.2010.091210 PMC2928963

[14] McEwan M, Lins RJ, Munro SK, Vincent ZL, Ponnampalam AP, et al (2009) Cytokine regulation during the formation of the fetal-maternal interface: focus on cell-cell adhesion and remodelling of the extra-cellular matrix. Cytokine Growth Factor Rev 20: 241–249. 19487153 10.1016/j.cytogfr.2009.05.004

[15] Powers C, DeFilippis V, Malouli D, Fruh K (2008) Cytomegalovirus immune evasion. Curr Top Microbiol Immunol 325: 333–359. 18637515 10.1007/978-3-540-77349-8_19

[16] van de Berg PJ, Heutinck KM, Raabe R, Minnee RC, Young SL, et al (2010) Human cytomegalovirus induces systemic immune activation characterized by a type 1 cytokine signature. J Infect Dis 202: 690–699. 20632887 10.1086/655472

[17] Scott GM, Chow SS, Craig ME, Pang CN, Hall B, et al (2012) Cytomegalovirus infection during pregnancy with maternofetal transmission induces a proinflammatory cytokine bias in placenta and amniotic fluid. J Infect Dis 205: 1305–1310. 22383678 10.1093/infdis/jis186

[18] Briana DD, Boutsikou M, Baka S, Papadopoulos G, Gourgiotis D, et al (2007) Perinatal plasma monocyte chemotactic protein-1 concentrations in intrauterine growth restriction. Mediators Inflamm 2007: 65032. 18274642 10.1155/2007/65032 PMC2234089

[19] Renaud SJ, Sullivan R, Graham CH (2009) Tumour necrosis factor alpha stimulates the production of monocyte chemoattractants by extravillous trophoblast cells via differential activation of MAPK pathways. Placenta 30: 313–319. 19201463 10.1016/j.placenta.2009.01.001

[20] Goser S, Ottl R, Brodner A, Dengler TJ, Torzewski J, et al (2005) Critical role for monocyte chemoattractant protein-1 and macrophage inflammatory protein-1alpha in induction of experimental autoimmune myocarditis and effective anti-monocyte chemoattractant protein-1 gene therapy. Circulation 112: 3400–3407. 16316965 10.1161/CIRCULATIONAHA.105.572396

[21] Haider S, Knofler M (2009) Human tumour necrosis factor: physiological and pathological roles in placenta and endometrium. Placenta 30: 111–123. 19027157 10.1016/j.placenta.2008.10.012 PMC2974215

[22] Chaiworapongsa T, Romero R, Tolosa JE, Yoshimatsu J, Espinoza J, et al (2002) Elevated monocyte chemotactic protein-1 in amniotic fluid is a risk factor for pregnancy loss. J Matern Fetal Neonatal Med 12: 159–164. 12530612 10.1080/jmf.12.3.159.164

[23] Renaud SJ, Postovit LM, Macdonald-Goodfellow SK, McDonald GT, Caldwell JD, et al (2005) Activated macrophages inhibit human cytotrophoblast invasiveness in vitro. Biol Reprod 73: 237–243. 15800179 10.1095/biolreprod.104.038000

[24] Toder V, Fein A, Carp H, Torchinsky A (2003) TNF-alpha in pregnancy loss and embryo maldevelopment: a mediator of detrimental stimuli or a protector of the fetoplacental unit? J Assist Reprod Genet 20: 73–81. 12688591 10.1023/A:1021740108284 PMC3455795

[25] Lopez H, Benard M, Saint-Aubert E, Baron M, Martin H, et al (2011) Novel model of placental tissue explants infected by cytomegalovirus reveals different permissiveness in early and term placentae and inhibition of indoleamine 2,3-dioxygenase activity. Placenta 32: 522–530. 21605903 10.1016/j.placenta.2011.04.016

[26] Robbins JR, Skrzypczynska KM, Zeldovich VB, Kapidzic M, Bakardjiev AI (2010) Placental syncytiotrophoblast constitutes a major barrier to vertical transmission of Listeria monocytogenes. PLoS Pathog 6: e1000732. 20107601 10.1371/journal.ppat.1000732 PMC2809766

[27] Cheeran MC, Hu S, Yager SL, Gekker G, Peterson PK, et al (2001) Cytomegalovirus induces cytokine and chemokine production differentially in microglia and astrocytes: antiviral implications. J Neurovirol 7: 135–147. 11517386 10.1080/13550280152058799

[28] Chan G, Guilbert LJ (2006) Ultraviolet-inactivated human cytomegalovirus induces placental syncytiotrophoblast apoptosis in a Toll-like receptor-2 and tumour necrosis factor-alpha dependent manner. J Pathol 210: 111–120. 16826536 10.1002/path.2025

[29] Vince G, Shorter S, Starkey P, Humphreys J, Clover L, et al (1992) Localization of tumour necrosis factor production in cells at the materno/fetal interface in human pregnancy. Clin Exp Immunol 88: 174–180. 1563104 10.1111/j.1365-2249.1992.tb03059.x PMC1554385

[30] Steinborn A, von Gall C, Hildenbrand R, Stutte HJ, Kaufmann M (1998) Identification of placental cytokine-producing cells in term and preterm labor. Obstet Gynecol 91: 329–335. 9491855 10.1016/s0029-7844(97)00680-7

[31] Dargan DJ, Douglas E, Cunningham C, Jamieson F, Stanton RJ, et al (2010) Sequential mutations associated with adaptation of human cytomegalovirus to growth in cell culture. J Gen Virol 91: 1535–1546. 20479471 10.1099/vir.0.018994-0 PMC3052722

[32] Katabuchi H, Yih S, Ohba T, Matsui K, Takahashi K, et al (2003) Characterization of macrophages in the decidual atherotic spiral artery with special reference to the cytology of foam cells. Med Electron Microsc 36: 253–262. 16228658 10.1007/s00795-003-0223-2

[33] Esplin MS, Peltier MR, Hamblin S, Smith S, Fausett MB, et al (2005) Monocyte chemotactic protein-1 expression is increased in human gestational tissues during term and preterm labor. Placenta 26: 661–671. 16085045 10.1016/j.placenta.2004.09.012

[34] Garcia-Lloret MI, Winkler-Lowen B, Guilbert LJ (2000) Monocytes adhering by LFA-1 to placental syncytiotrophoblasts induce local apoptosis via release of TNF-alpha. A model for hematogenous initiation of placental inflammations. J Leukoc Biol 68: 903–908. 11129659

[35] Sadowsky DW, Adams KM, Gravett MG, Witkin SS, Novy MJ (2006) Preterm labor is induced by intraamniotic infusions of interleukin-1beta and tumor necrosis factor-alpha but not by interleukin-6 or interleukin-8 in a nonhuman primate model. Am J Obstet Gynecol 195: 1578–1589. 17132473 10.1016/j.ajog.2006.06.072

[36] Gendron RL, Nestel FP, Lapp WS, Baines MG (1990) Lipopolysaccharide-induced fetal resorption in mice is associated with the intrauterine production of tumour necrosis factor-alpha. J Reprod Fertil 90: 395–402. 2250238 10.1530/jrf.0.0900395

[37] Bauer S, Pollheimer J, Hartmann J, Husslein P, Aplin JD, et al (2004) Tumor necrosis factor-alpha inhibits trophoblast migration through elevation of plasminogen activator inhibitor-1 in first-trimester villous explant cultures. J Clin Endocrinol Metab 89: 812–822. 14764800 10.1210/jc.2003-031351

[38] Huber AV, Saleh L, Bauer S, Husslein P, Knofler M (2006) TNFalpha-mediated induction of PAI-1 restricts invasion of HTR-8/SVneo trophoblast cells. Placenta 27: 127–136. 16338458 10.1016/j.placenta.2005.02.012

[39] Chan G, Stinski MF, Guilbert LJ (2004) Human cytomegalovirus-induced upregulation of intercellular cell adhesion molecule-1 on villous syncytiotrophoblasts. Biol Reprod 71: 797–803. 15140794 10.1095/biolreprod.104.028118

[40] Lockwood CJ, Oner C, Uz YH, Kayisli UA, Huang SJ, et al (2008) Matrix metalloproteinase 9 (MMP9) expression in preeclamptic decidua and MMP9 induction by tumor necrosis factor alpha and interleukin 1 beta in human first trimester decidual cells. Biol Reprod 78: 1064–1072. 18276934 10.1095/biolreprod.107.063743 PMC3045968

[41] Leisser C, Saleh L, Haider S, Husslein H, Sonderegger S, et al (2006) Tumour necrosis factor-alpha impairs chorionic gonadotrophin beta-subunit expression and cell fusion of human villous cytotrophoblast. Mol Hum Reprod 12: 601–609. 16896069 10.1093/molehr/gal066

[42] Maidji E, McDonagh S, Genbacev O, Tabata T, Pereira L (2006) Maternal antibodies enhance or prevent cytomegalovirus infection in the placenta by neonatal Fc receptor-mediated transcytosis. Am J Pathol 168: 1210–1226. 16565496 10.2353/ajpath.2006.050482 PMC1606573

[43] Koi H, Zhang J, Makrigiannakis A, Getsios S, MacCalman CD, et al (2002) Syncytiotrophoblast is a barrier to maternal-fetal transmission of herpes simplex virus. Biol Reprod 67: 1572–1579. 12390890 10.1095/biolreprod.102.004325

[44] Koi H, Zhang J, Parry S (2001) The mechanisms of placental viral infection. Ann N Y Acad Sci 943: 148–156. 11594535 10.1111/j.1749-6632.2001.tb03798.x

[45] Yamamoto-Tabata T, McDonagh S, Chang HT, Fisher S, Pereira L (2004) Human cytomegalovirus interleukin-10 downregulates metalloproteinase activity and impairs endothelial cell migration and placental cytotrophoblast invasiveness in vitro. J Virol 78: 2831–2840. 14990702 10.1128/JVI.78.6.2831-2840.2004 PMC353759

[46] Bilban M, Tauber S, Haslinger P, Pollheimer J, Saleh L, et al (2010) Trophoblast invasion: assessment of cellular models using gene expression signatures. Placenta 31: 989–996. 20850871 10.1016/j.placenta.2010.08.011

[47] Carter AM (2007) Animal models of human placentation–a review. Placenta 28 Suppl A: S41–47. 10.1016/j.placenta.2006.11.002 17196252

[48] Jurak I, Brune W (2006) Induction of apoptosis limits cytomegalovirus cross-species infection. EMBO J 25: 2634–2642. 16688216 10.1038/sj.emboj.7601133 PMC1478185

[49] Tabata T, Petitt M, Fang-Hoover J, Rivera J, Nozawa N, et al.. (2012) Cytomegalovirus Impairs Cytotrophoblast-Induced Lymphangiogenesis and Vascular Remodeling in an in Vivo Human Placentation Model. Am J Pathol. 10.1016/j.ajpath.2012.08.003 PMC3483806 22959908

[50] Stanton RJ, Baluchova K, Dargan DJ, Cunningham C, Sheehy O, et al (2010) Reconstruction of the complete human cytomegalovirus genome in a BAC reveals RL13 to be a potent inhibitor of replication. J Clin Invest 120: 3191–3208. 20679731 10.1172/JCI42955 PMC2929729

[51] Faye A, Pornprasert S, Dolcini G, Ave P, Taieb J, et al (2005) Evaluation of the placental environment with a new in vitro model of histocultures of early and term placentae: determination of cytokine and chemokine expression profiles. Placenta 26: 262–267. 15708128 10.1016/j.placenta.2004.08.005

